# High quality factor confined Tamm modes

**DOI:** 10.1038/s41598-017-04227-1

**Published:** 2017-06-20

**Authors:** C. Symonds, S. Azzini, G. Lheureux, A. Piednoir, J. M. Benoit, A. Lemaitre, P. Senellart, J. Bellessa

**Affiliations:** 1 Univ Lyon, Université Claude Bernard Lyon 1, CNRS, Institut Lumière Matière, F-69622 Lyon, France; 2 Centre de Nanosciences et Nanotechnologies, CNRS Université Paris-Saclay, Route de Nozay, F-91460 Marcoussis, France

## Abstract

We demonstrate that quality factors up to 5000 can be obtained in Tamm-like hybrid metal/semiconductor structures. To do this, a Bragg mirror is covered by a thin transparent layer and a metallic film. The reduced losses of these modes are related to an intermediate behavior between conventional Tamm plasmon and Bragg modes lying deeper in the semiconductor medium. One of the most striking features of this approach is that these super Tamm modes can still be spatially confined with the metal. Confinement on micrometric scale is experimentally demonstrated. The simplicity and versatility of high-Q mode control by metal structuration open perspectives for lasing and polaritonic applications.

## Introduction

The spatial confinement of optical modes is a key point for emission control in nanostructures as it directly affects the Purcell factor^1, 2^. Plasmonic structures present a very small mode volume and allow high emission rate enhancement up to 1000^3^. Nevertheless their relatively low quality factor can be a drawback for lasing or for strong light matter coupling applications with semiconductors^4^. On the other hand, dielectric micro/nano structures have larger mode volumes but very high quality factors^5–7^. They are particularly well suited for lasing, and polariton Bose-Einstein condensation has been evidenced in these systems. Nevertheless, the mode shape remains difficult to control or manipulate. Methods based on direct milling of the structures^8, 9^ or mesa etching^10, 11^ have been developed and have proven their efficiency at the cost of complex technological processes. However new developments in lasers, such as orbital angular momentum microlasers^12^, or in polariton physics^13^ (e.g. 2D lattices for polariton band engineering^14^, coupled cavities for Josephson effect)^15^ require an increased complexity in term of control of the mode confinement. The hybrid metal dielectric optical Tamm structures have been proposed to overcome this issue^16, 17^. These modes are formed at the interface between a Distributed Bragg Reflector (DBR) and a metal film^18, 19^. The shape of the mode is easily controlled by a structuration of the metallic part allowing an easy realization of complex geometries, while the losses are reduced due to the dielectric part of the system. Strong coupling with quantum well exciton^20, 21^ as well as single photon emission control^22^ or lasing action^23–25^ has been evidenced in these structures. Nevertheless, the quality factor of the Tamm mode remains of the order of one thousand, which is too low for Bose condensation in strong coupling^26^ and do not allow a strong reduction of lasing threshold^23, 27^.

In this paper, we propose a structure based on a geometry similar to the Tamm plasmon and allowing at the same time an increased quality factor and a confinement purely achieved by metal patterning. For this purpose, a thin dielectric film is inserted between the DBR and the metal in a conventional Tamm plasmon structure. The electromagnetic structure of the mode is then strongly modified, with a delocalization in the dielectric DBR. Playing on the spacer thickness it is possible to obtain a mode with better quality factor than both the DBR mode and the Tamm mode. To unambiguously separate this mode from Tamm plasmon (TP) mode, we will refer to it as super Tamm (ST) mode. We will first describe theoretically and experimentally the main properties of 2-dimensional ST modes. We will then demonstrate that this mode can be confined by a proper patterning of the metallic layer.

## Results and Discussion

**Figure 1:**
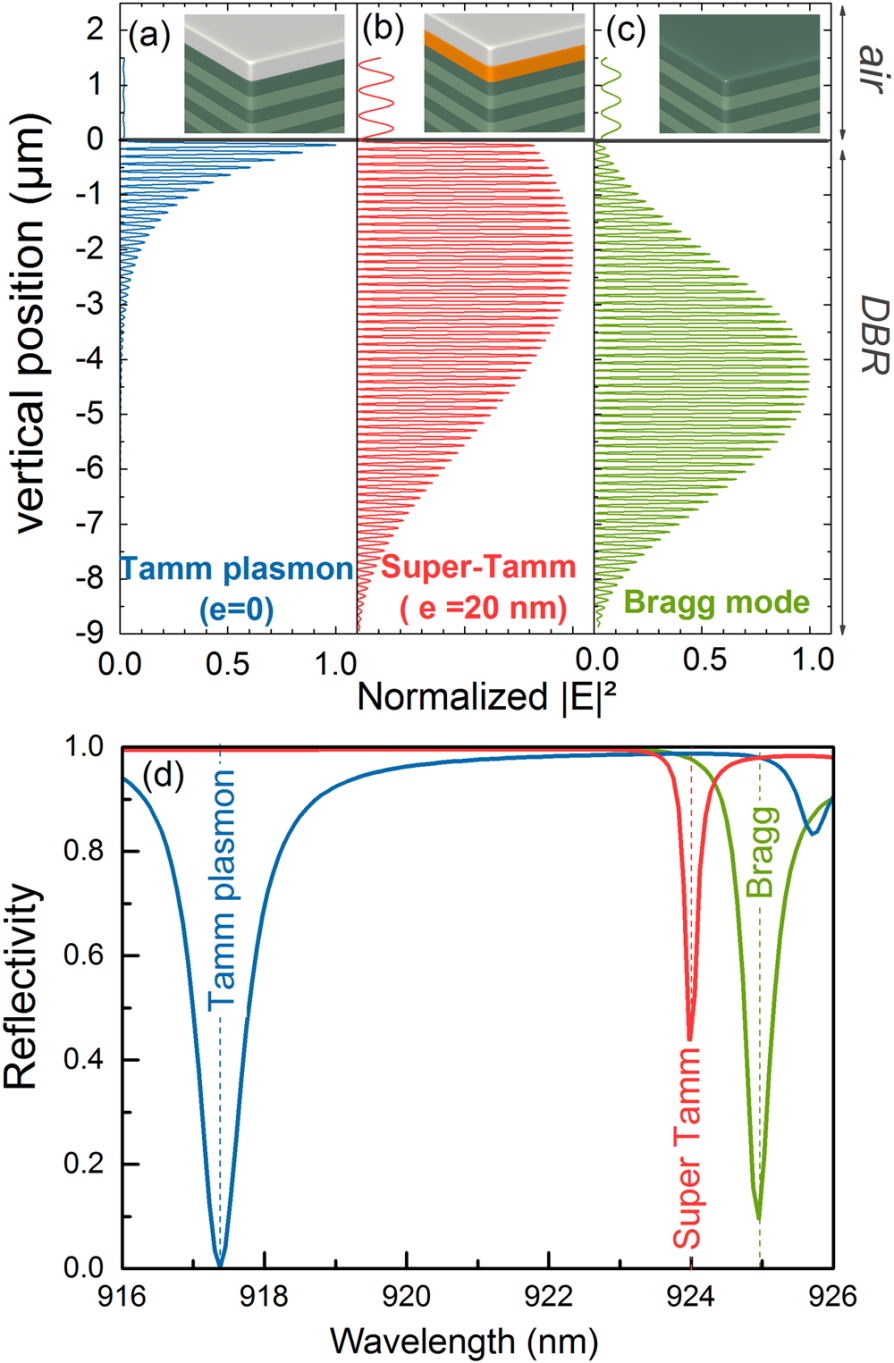
Spatial repartition of the normalized squared electric field calculated for the (**a**) Tamm plasmon mode (λ = 917.4 nm); (**b**) super Tamm mode (λ = 924 nm); (**c**) first Bragg mode (λ = 925 nm). (**d**) Calculated reflectivities of Tamm plasmon, super Tamm and bare DBR structures. The Tamm structures comprise a 45 nm thick silver layer deposited on the DBR, and a 20 nm low refractive index layer (n = 1.485) is inserted between the metal and the DBR to form the super Tamm mode.

Transfer matrix simulations were performed to calculate the squared electric field and the reflectivity associated to Tamm structures including a dielectric spacer of variable thickness. All the structures comprise the same 65 pairs GaAs/AlGaAs DBR centered at 880 nm. A 45 nm silver film is added on the top of the DBR to form a TP structure, and a 20 nm transparent spacer (n = 1.485) is inserted between the metal and the DBR to form a ST structure. The normalized squared electric fields associated to the TP and SP modes are plotted in Fig. 1(a,b). The normalized squared electric field of the bare DBR first Bragg mode (i.e. reflectivity minimum on the low energy side of the stopband) is also presented in Fig. 1c. The TP mode presents a characteristic surface-mode shape, with an exponential decrease of its envelope starting from the interface. Its decay length is 0.9 µm, much smaller than the DBR thickness (8.9 µm), indicating that in this case the TP quality factor is limited by the metal rather than by the DBR reflectivity. The DBR mode envelope presents a Gaussian-like shape centered in the middle of the DBR. The ST electric field profile is halfway between a TP mode and a Bragg mode: it is still confined at the metal/dielectric interface, but then expands in the DBR with a truncated Gaussian-like shape.

The reflectivity of the TP, ST and bare DBR structures are shown in Fig. 1d, each presenting a dip associated to a resonance. The linewidth of the ST mode is 0.18 nm (Q = 5100), smaller than the TP and Bragg mode widths (0.78 nm − Q = 1170 and 0.42 nm − Q = 2180, respectively). It has to be noted that the dip in the calculated reflectivity spectra is shallower for super Tamm modes than for TP and Bragg modes, indicating that the coupling of the planar incident wave to the mode is smaller for ST mode. For an emitter inserted in the Tamm or ST structure, this can be related to the extraction of light.

**Figure 2:**
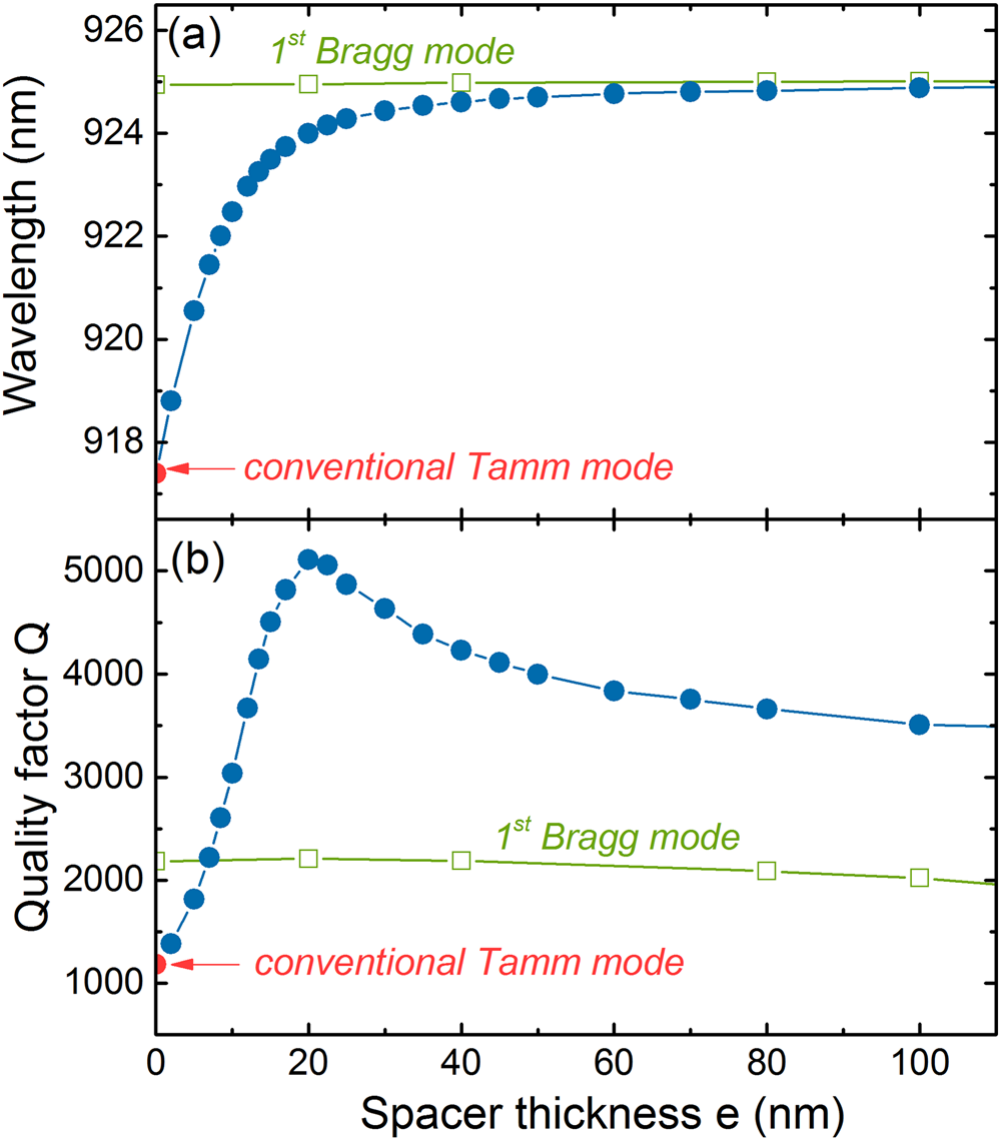
Variation of (**a**) resonance wavelength and (**b**) quality factor with the spacer thickness calculated by the transfer matrix method. The spacer (n = 1.485) is either inserted between a 65 pairs DBR and a 45 nm thick silver layer (blue circles) or deposited on a 65 pairs DBR without metal (green open squares).

To go further in the analysis of ST modes, the variation of its resonance wavelength and quality factor has been plotted as a function of the spacer thickness e in Fig. 2 (bue circles). For e = 0, the values correspond to the ones of the TP mode. When the spacer thickness is increased, the resonance wavelength shifts to higher values and gradually approaches the one corresponding to the DBR’s first Bragg mode, as previously observed when modifying the last GaAs layer thickness in a Tamm structure^28^. At the same time the quality factor strongly increases up to 5100 for e = 20 nm, then slowly decreases from this value to a value of Q = 4000 for thicker spacers. This value remains always larger than the one associated to both conventional Tamm plasmon and Bragg modes. For comparison, the same dielectric thickness was also added on the DBR without metal on top (green open squares in Fig. 2(a,b)). In this case the dielectric film has no impact on the first Bragg mode resonance wavelength, and leads to a small modification (less than 10%) of its quality factor^29^ which cannot explain by itself the 5-fold enhancement obtained for the ST mode.

To give a simplified physical picture for the quality factor improvement in ST structures, we can consider the different losses channels. In Tamm plasmon structures losses are mainly associated to the metal (imaginary part of the dielectric constant), and in a bare DBR to the coupling to radiative modes at the upper and lower interfaces. Compared to the Tamm plasmon, the super Tamm is less bounded to the interface. The reduction of the losses can thus be associated to the reduction of the electric field extension in the metal compared to the dielectric part, in a similar way as other low losses surface plasmon-like modes (e.g. long range surface plasmon)^30^. Moreover, compared to the first DBR mode the ST field maximum is farther from the bottom interface which reduces its radiative losses. A qualitative indication of the radiative losses reduction can be obtained when dividing the electric field in the last DBR bottom layer by the total internal electric field in the structure. This ratio is 0.12% for the first Bragg mode of the bare DBR, and 0.045% for a ST mode formed with a 20 nm thick dielectric spacer, indicating that the losses are higher for the Bragg mode than for the ST mode. The reduction of the field extension in the metal can be estimated in the same way: the fraction of the electric field lying in the metal is 0.82% for the Tamm and decreases down to 0.09% for the ST mode.

**Figure 3:**
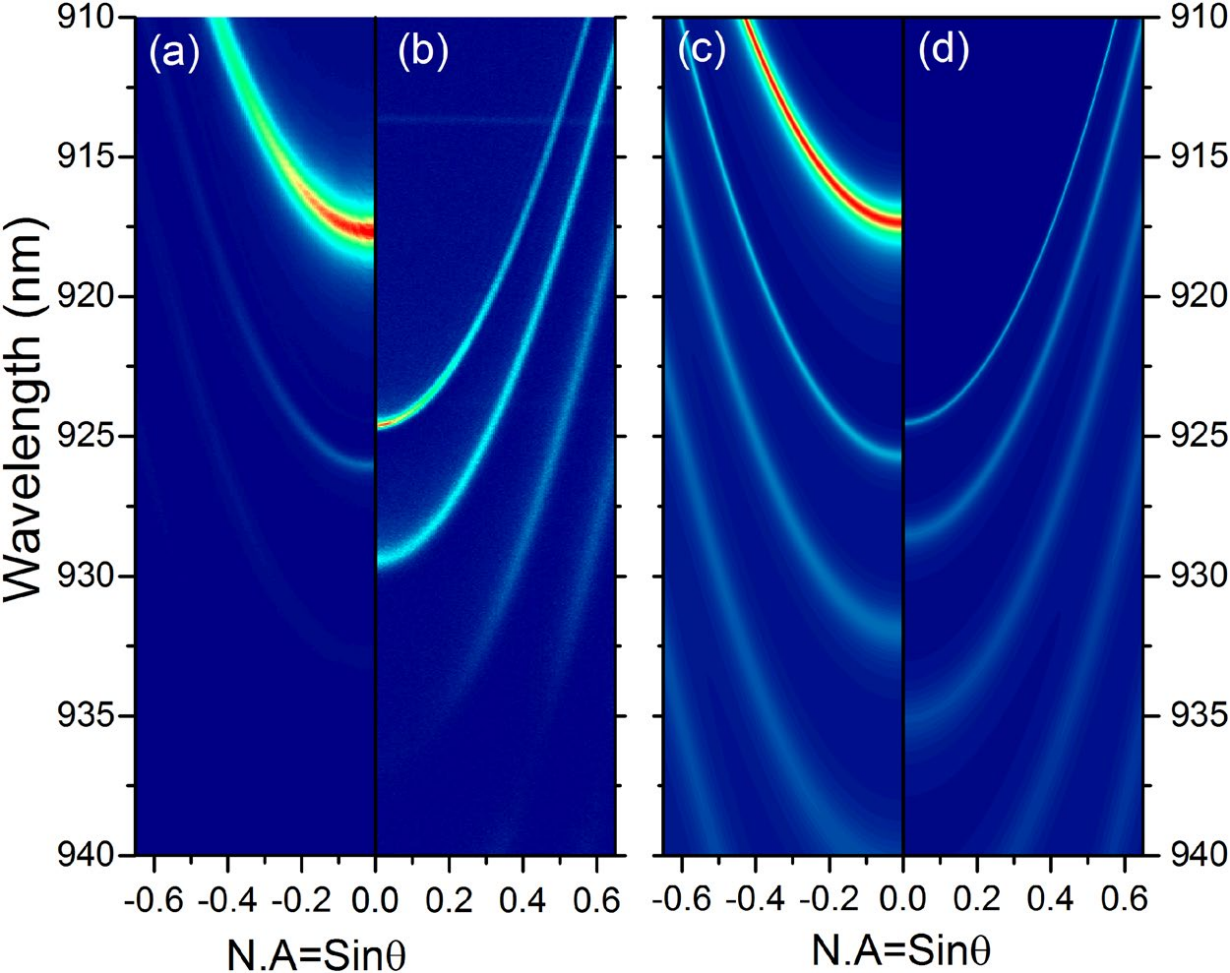
Experimental emission dispersion relations recorded on the (**a**) Tamm plasmon and (**b**) super Tamm plasmon (e = 35 nm) areas. (**c**) and (**d**) corresponds to reflectivities transfer matrix calculations on a Tamm and super Tamm structure, respectively.

In order to experimentally evidence the formation of ST modes, a 65 pairs GaAs/AlGaAs DBR containing InAs/GaAs quantum dots (QDs) was fabricated (see Methods). These QDs have a broadband emission around 920 nm. Part of this DBR is covered by a 35 nm thick PMMA layer (n = 1.485). A 45 nm silver layer was deposited in both areas of the DBR, i.e. directly on the last GaAs layer to form a TP mode, and on the PMMA layer to form a ST mode. Photoluminescence experiments were performed by exciting the sample with a continuous-wave Ti:Sapphire laser focused on the surface with a microscope objective (numerical aperture NA = 0.75). The emitted light is collected by the same objective and sent to a spectrometer associated to a CCD camera. The Fourier plane of the objective can be imaged on the spectrometer entrance slit in order to record the emission dispersion relation. The dispersion relation of the TP mode is presented in Fig. 3a, where the vertical axis corresponds to the wavelength, and the horizontal axis to the sinθ of the collected emission. The TP mode is centered at 917.6 nm, and a quality factor of Q = 870 can be extracted from its linewidth. The weaker parabolas lying at higher wavelengths correspond to higher-orders Tamm modes. When shifting to the ST area (where the PMMA layer is inserted between the silver and the GaAs) the dispersion relation is strongly modified, as shown in Fig. 3b. In this case the ST mode is shifted to higher wavelength, as well as the higher order ST modes. More importantly, the ST linewidth is strongly reduced and can be associated to a quality factor of Q = 4860. This corresponds to an enhancement of 5.5 compared to the TP mode. These dispersion extracted from experimental results in emission are in good agreement with the one deduced from reflectivity of the structures calculated by the transfer matrix method (Fig. 3(c,d)). It should be noted that the predicted Q factor of the Tamm plasmon is 25% higher than one measured experimentally. This discrepancy could be attributed to higher losses in our evaporated silver film. Moreover the experimental Q-enhancement (5) is lower than the experimental one (5.5). This could be explained by the fact, that as ST modes are less sensitive to metal losses than TP modes, an underestimation of the real metal losses leads to a higher calculation-experiment discrepancy for the TP than for the ST mode.

**Figure 4:**
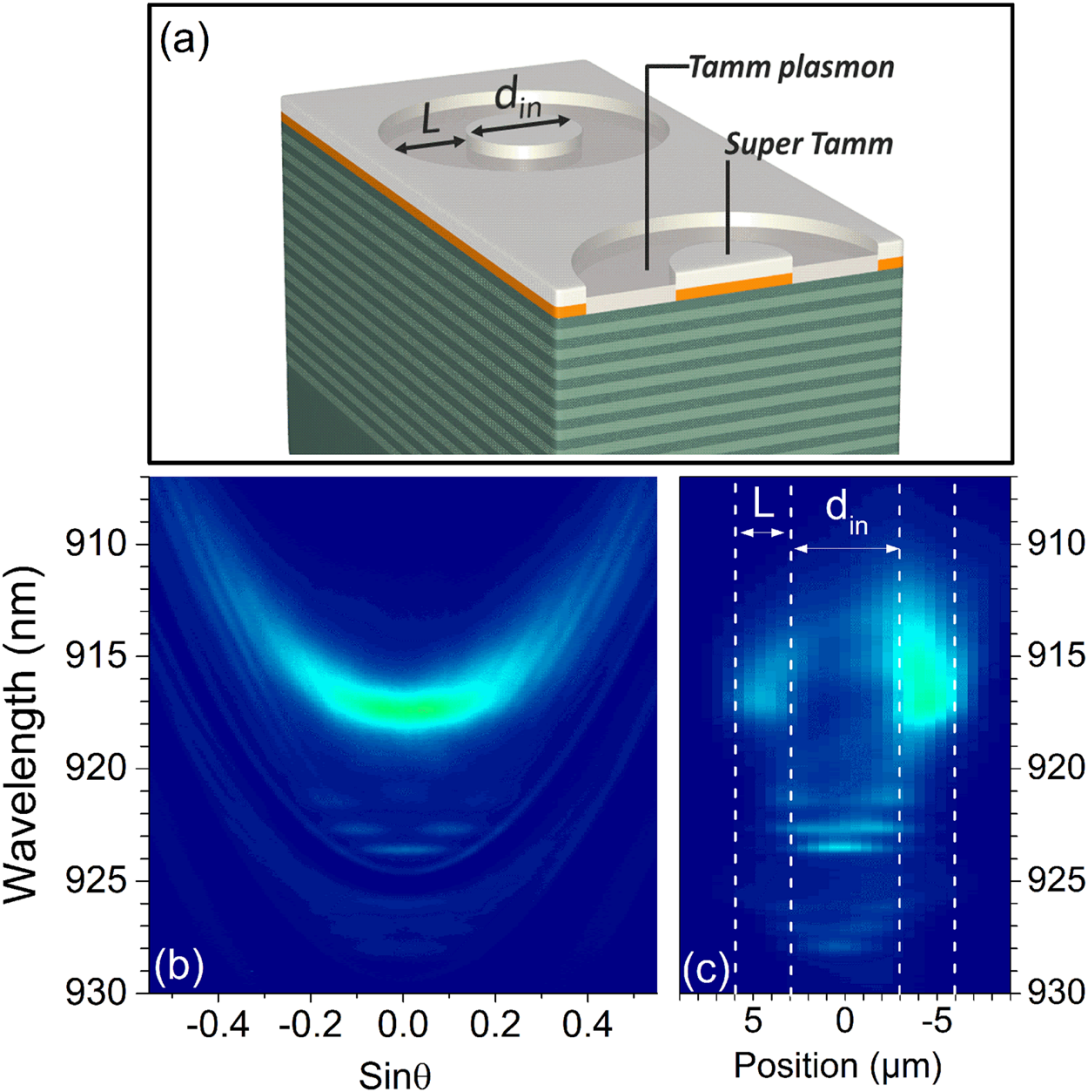
(**a**) Schematic representation of the sample evidencing the DBR, the PMMA resist layer (e = 35 nm) and the 45 nm silver layer. Rings of inner diameter d_in_ and arm width L are defined in the PMMA layer. (**b**) Experimental emission dispersion relation recorded on a ring of inner diameter d_in_ = 6 µm. (**c**) Wavelength-resolved emission as a function of the position along the diameter of the ring.

The quality factor of ST mode can thus be as high as 5000. A very important aspect of this approach is that it preserves the confinement potentiality associated to metal patterning. Due to the wavelength shift between super-Tamm and Tamm modes, the ST mode can be confined if surrounded by a TP region. To demonstrate this point, ring structures of various inner diameter d_in_ and arm width L have been defined in the PMMA resist by e-beam lithography (Fig. 4a). In the inner and outer parts of the ring the 35 nm thick PMMA layer remains unchanged, leading to the formation of a ST mode when depositing the metal. On the contrary in the ring arm itself the PMMA is removed thus leading to the formation of a conventional TP mode in this area.

The emission dispersion relation recorded on a ring structure (d_in_ = 6 µm, L = 3 µm) is presented in Fig. 4b, where the vertical axis correspond to the wavelength and the horizontal axis to the detection angle sinθ. A spatially and spectrally resolved image of the sample is also presented in Fig. 4c, where the vertical axis corresponds to the wavelength and the horizontal axis to the direction along a vertical cross section on the structure, recorded by collecting all the emission in the objective numerical aperture. The bright emission lying around 917 nm corresponds to the TP mode, and emanates from the ring itself as seen in Fig. 4c. The parabolas associated to the first and second order ST modes are also visible in Fig. 4b around 924 nm and 929 nm. The most remarkable features in this dispersion relation are the emission lobes located at lower wavelength starting from the first and second order ST modes. These emission lobes correspond to a discretization of the emission, associated to a 3D confinement. This is confirmed in the spatial/spectral image of Fig. 4c, where the confined emission lobes can indeed be attributed to the central area of the sample, i.e the area where the ST mode is laterally confined by the TP ring.

**Figure 5:**
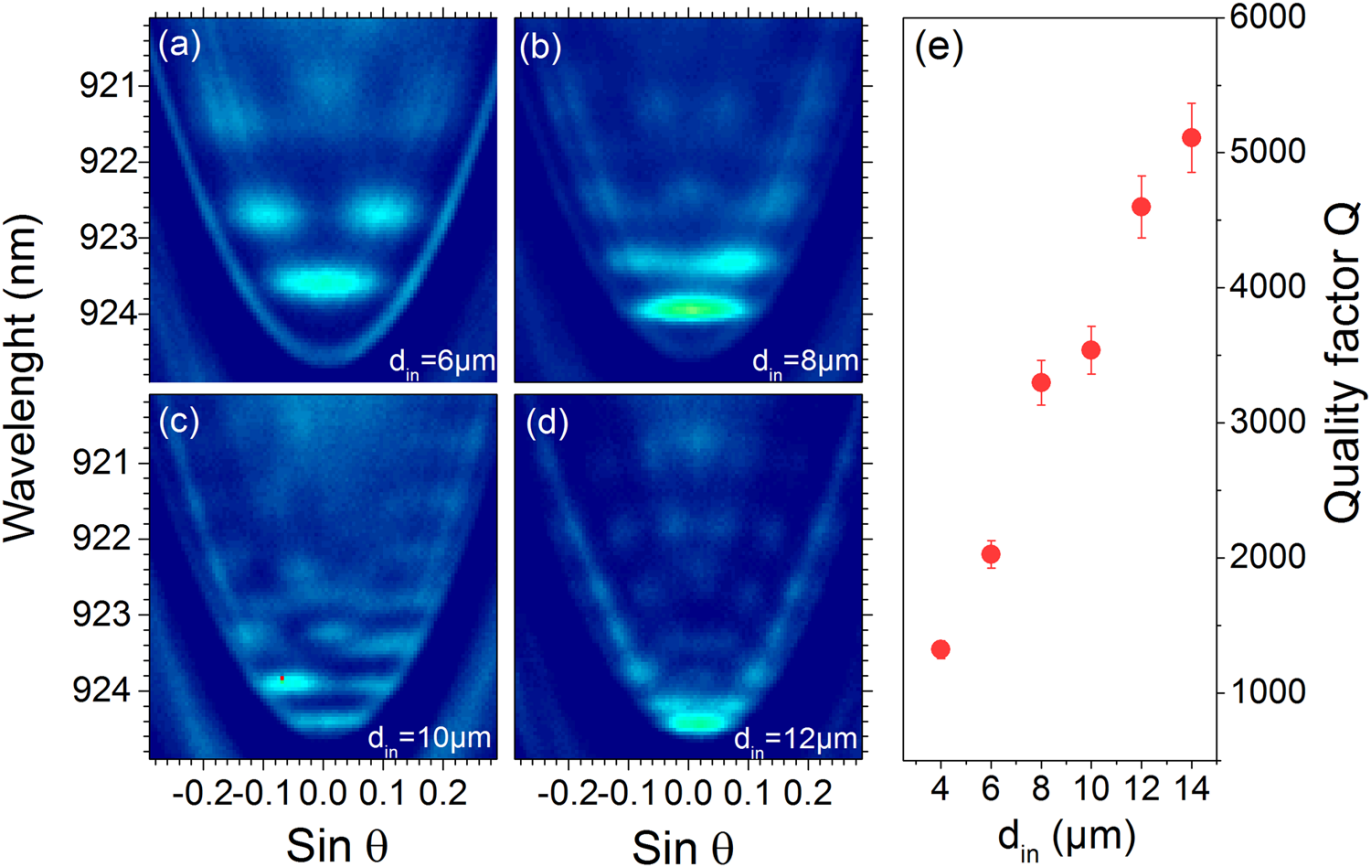
(**a**–**d**) Experimental dispersion relations of the emission recorded for rings with inner diameter d_in_ of 6, 8, 10, and 12 µm. (**e**) Variation of the confined super Tamm fundamental mode quality factor as a function of d_in_.

The dispersion relations recorded for rings having inner diameters of 6, 8, 10 and 12 µm are represented Fig. 5a–d. Confined ST modes are visible in each of these dispersion relations. When increasing the inner diameter the confined mode splitting decreases, consistent with what is expected when increasing the confinement volume. The variation of the fundamental confined ST mode quality factor with d_in_ is plotted in Fig. 5e. The quality factor decreases with the size of the inner ring down to 1300 for a 4 µm disk. For a 8 µm disk the Q factor is 3200. A way to make the confinement more efficient could be to increase the ST confinement energy, i.e. the TP/ST energy mismatch.

## Conclusion

In conclusion we have shown theoretically and experimentally that super Tamm modes, exhibiting properties halfway between Tamm and Bragg modes, could be obtained in hybrid metal/dielectric structures. The insertion of a thin transparent layer between the DBR and the metal produces a delocalization of the electric field in the DBR, and leads to quality factors as high as 5000, which was confirmed experimentally. Nevertheless the mode remains attached to the metallic surface maintaining the high versatility associated to the confinement with metal structuration. We have indeed demonstrated that super Tamm modes could be confined by a proper patterning of the metallic layer only, while maintaining a relatively good quality factor.

The simple confinement as well as the low losses of these structures could be of crucial interest for the manipulation of exciton polaritons. For this purpose the ST could be directly used to hybridize QW exciton or coupled to a cavity polaritons^31, 32^. This could lead to new functionalities associated with tailored properties of Bose condensates^33^. The ST could also be exploited for the realization of original laser structures inducing angular momentum^12^. Metallic geometries like rings or spiral could allow and efficient control of the moment of the mode^34^.

## Methods

Transfer matrix methods were used to calculate the reflectivity and electric field of the structures. For the simulations, the DBR centered at 880 nm was formed by 65 pairs of GaAs (n = 3.57, e = 62 nm) and Al_95_Ga_05_As (n = 2.98, e = 74.5 nm) layers on a GaAs substrate. The spacer consists in a transparent layer of refractive index 1.485. A 45 nm silver layer is added either directly on the last GaAs layer to simulate a Tamm mode, or on the spacer to simulate the super Tamm mode. The theoretical quality factors where estimated by a Lorentzian fit of the calculated reflectivity spectra. The experimental sample was grown by Molecular Beam Epitaxy (MBE) on a GaAs substrate. The difference between the experimental and modeled sample is that during the growth three pairs of highly dense self-assembled InAs/GaAs quantum dots where embedded in the DBR: one pair is located in the penultimate GaAs layer, and two pairs in the topmost GaAs layer. It should be noted that these QDs layers represents typically 4% of the GaAs layer thickness, and exhibit a very close refractive index (n_InAs_ = 3.7 @ 880 nm): The GaAs layer refractive index variation due to the presence of InAs is thus about 0.1%. It is therefore reasonable not to take these QDs layers into account in the transfer matrix simulations. A 35 nm thick layer of poly(methylmethacrylate) was then spin-coated on the top of the sample, and annealed at 170° for 10 min. In order to compare Tamm and super Tamm modes, the PMMA resist layer was then removed by electron beam lithography (e-beam) on large circular areas (diameter 50 µm). In order to confine the super Tamm modes, on another region of the same sample the PMMA layer was removed by e-beam lithography on annular patterns of various inner and outer diameters. A 45 nm thick silver layer was then deposited by sputtering on the whole sample surface: in the areas where the PMMA was removed (Tamm areas) and on the areas where the PMMA layer remained (super-Tamm or confined super-Tamm areas). Photoluminescence experiments were performed at 77 K. The devices are excited from the top trough the silver layer with a continuous-wave Ti:Sapphire laser at 760 nm, focused on a 1.8 µm diameter spot on the sample using a microscope objective (numerical aperture NA = 0.75). The emitted light is collected by the same objective and sent to a spectrometer associated to a CCD camera. The Fourier plane of the objective can be imaged on the spectrometer entrance slit in order to record the emission dispersion relation. The experimental quality factors were estimated by a Lorentzian fit of the dispersion relation at k = 0. When removing the Fourier lens from the optical path, the sample surface is imaged on the vertical entrance slit of the spectrometer which results in a spatially (vertical axis) and spectrally (horizontal axis) resolved image on the CCD.
